# Prenatal anxiety, maternal stroking in infancy, and symptoms of emotional and behavioral disorders at 3.5 years

**DOI:** 10.1007/s00787-016-0886-6

**Published:** 2016-07-27

**Authors:** Andrew Pickles, Helen Sharp, Jennifer Hellier, Jonathan Hill

**Affiliations:** 1 Biostatistics Department, Institute of Psychiatry, Psychology and Neuroscience, King’s College, London, UK; 2 Institute of Psychology, Health and Society, University of Liverpool, Liverpool, UK; 3 School of Psychology and Clinical Language Sciences, University of Reading, Reading, UK

**Keywords:** Fetal programing, Prenatal anxiety, Tactile stimulation, Epigenetics, Emotional, Behavioral disorders

## Abstract

Animal findings of long-term effects of maternal behaviors mediated via altered GR gene expression will, if translated into humans, have far reaching implications for our understanding of child and adolescent psychopathology. We have previously shown that mothers’ self-reported stroking of their infants modifies associations between prenatal depression and anxiety and child outcomes at 29 weeks and 2.5 years. Here, we examine whether the effect of early maternal stroking is evident at 3.5 years, and in a much larger sample than in previous publications. A general population sample of 1233 first-time mothers completed anxiety measures at 20 weeks gestation, 865 reported on infant stroking at 9 weeks, and 813 on child symptoms at 3.5 years. Maternal stroking moderated the association between pregnancy-specific anxiety and internalizing (*p* = 0.010) and externalizing (*p* = 0.004) scores, such that an effect of PSA to increase symptoms was markedly reduced for mothers who reported high levels of stroking. There was no effect of maternal stroking on general anxiety. The findings confirm the previously reported effect of maternal stroking, and in a much larger sample. They indicate that there are long-term effects of early maternal stroking, modifying associations between prenatal anxiety and child emotional and behavioral symptoms.

**Electronic supplementary material:**

The online version of this article (doi:10.1007/s00787-016-0886-6) contains supplementary material, which is available to authorized users.

## Introduction

In animal models, prenatal stress causes long lasting ‘fetal programing’ increases in anxiety and depression type behaviors and hypothalamo-pituitary axis (HPA) reactivity, mediated via decreased hippocampal glucocorticoid receptor (GR) gene expression [1]. Rat mother licking and grooming (LG), and arched back nursing of offspring cause long lasting decreases in anxiety behaviors and HPA axis reactivity mediated via increased GR gene expression [2]. Many of the effects of LG found to mediate the association with GR gene expression can also be produced experimentally by stroking rat pups with a brush [3]. In studies of humans, maternal anxiety and depression during pregnancy have been found to predict childhood disruptive behavior problems after controlling for postnatal environmental factors [4, 5], and prenatal maternal anxiety predicts persistence from childhood to adolescence [6].

Equally some studies have failed to identify associations between prenatal anxiety and later outcomes. One possible explanation is that measures of general anxiety reflect a mix of long standing and current anxiety, and so do not adequately capture the mother’s psychological state during pregnancy [7]. It may therefore be that measures of pregnancy-specific stress, focusing on worries about the pregnancy and the fetus, are better than measures of generalized psychological distress for predicting developmental outcomes. For example, in a study with measures of maternal anxiety at five time points during pregnancy, pregnancy-specific anxiety, but not general anxiety predicted maternal report of negative emotionality at age 2 [7] and poorer performance on executive function tasks at ages 6–9 [8].

Animal studies have also shown sex differences in physiological, gene expression, and behavioral responses to prenatal stress [9–11]. Generally, the increased anxiety and depression type behaviors and HPA axis changes are seen only in females [12, 13]. Sex differences have also been reported in human studies, where associations between low birth weight [14, 15], prenatal anxiety [16], prenatal depression [17], and adolescent depression, only in girls, have been found.

Based on the possibility that the effects of LG in animal models arise from signaling associated with tactile stimulation, we examined the role of infant stroking by mothers. Using an intensively assessed sub-sample (*N* = 271) of the Wirral Child Health and Development Study, we showed that maternal stroking over the first weeks of life modified the association between prenatal depression and physiological and behavioral reactivity at 7 months [18]. Increasing maternal depression was associated with decreasing vagal withdrawal, a measure of physiological adaptability, and with increasing negative emotionality, only in the presence of low maternal stroking. In other words, high maternal stroking eliminated the associations between prenatal depression and early developmental outcomes. Subsequently, we found that that maternal stroking modifies, in similar fashion, the association between prenatal anxiety and internalizing symptoms at 2.5 years, specifically in girls [19]. The aim of this study was to examine whether the effect of maternal stroking is evident over a longer period and in a much larger sample than in our previous publications. We also compare the effects of general anxiety and pregnancy-specific anxiety, and test for sex differences.

## Methods

### Study design and Sample

The participants were members of the Wirral Child Health and Development Study, a prospective epidemiological longitudinal study starting in pregnancy with follow-up over several assessment points during infancy up to when the children were 3.5 years. This uses a two-stage stratified design in which a consecutive general population sample (the ‘extensive’ sample) is used to generate a smaller ‘intensive’ sample stratified by psychosocial risk and both are followed in tandem. This enables intensive measurement to be employed efficiently with the stratified subsample, while weighting back to the extensive sample enables general population estimates to be derived. The analyses presented here focus on the larger extensive sample which was identified from consecutive first-time mothers who booked for antenatal care at 12 weeks gestation between 12/02/2007 and 29/10/2008. The booking clinic was administered by the Wirral University Teaching Hospital which was the sole provider of universal prenatal care on the Wirral Peninsula, a geographical area bounded on three sides by water. Socioeconomic conditions on the Wirral range between the deprived inner city and affluent suburbs, but with very low numbers from ethnic minorities.

The study was introduced to the women at 12 weeks of pregnancy by clinic midwives who asked for their agreement to be approached by study research midwives when they attended for ultrasound scanning at 20 weeks gestation. Ethical approval for the study was granted by the Cheshire North and West Research Ethics Committee on the 27th June 2006, reference number 05/Q1506/107, and has therefore been performed in accordance with the ethical standards laid down in the 1964 Declaration of Helsinki and its later amendments. After obtaining written informed consent, the study midwives administered questionnaires. Of those approached by study midwives, 68.4 % gave consent and completed the measures, yielding an extensive sample of 1233 mothers with surviving singleton babies.

**Fig. 1:**
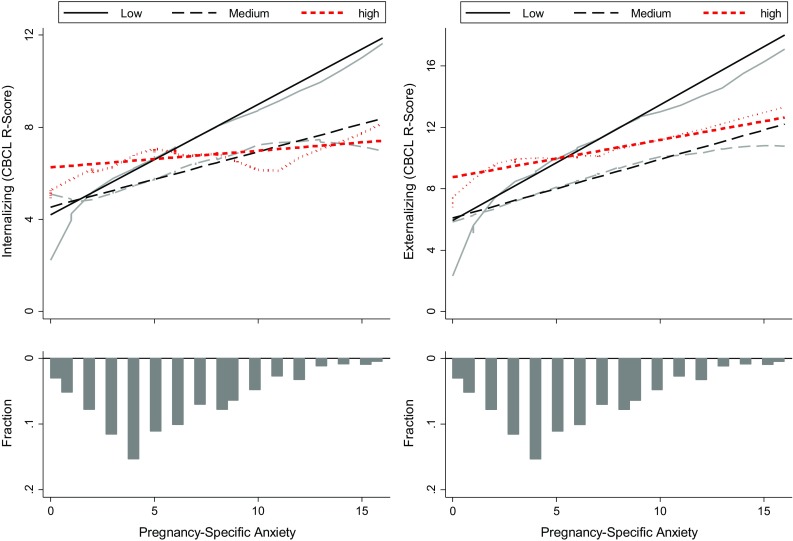
Locally weighted scatterplot smoothing (LOWESS) plots, and regression lines showing the association between pregnancy-specific anxiety and internalizing and externalizing symptoms, contrasting children of mothers in low (*black solid lines*), medium (*black dotted lines*), and high (*red dotted lines*) tertile stroking groups. The *bars* indicate the distributions of the pregnancy-specific anxiety scores

Numbers recruited to the extensive sample and followed up for the measures used in this analysis are shown in Fig. 1 of the Online Data Supplement. Data were available on all 1233 from birth records and for anxiety measures at 20 weeks of pregnancy (‘20 weeks gestation’) and on 865 mothers for infant stroking at 9.3 (sd 3.6) weeks (‘9 weeks’). The composite measure of postnatal anxiety was made up of data from 819 mothers. Child outcome data were gathered from 813 when the children were 41.80 (sd 2.3)-months-old (‘3.5 years’).

### Assessment of maternal anxiety and depression

Pregnancy anxiety was measured by The Pregnancy-Specific Anxiety Scale [20] at the 20 week initial assessment. Mothers completed the scale by responding to the question “how have you felt about being pregnant in the past week including today?” They were asked to rate four items each on a 5-point scale (where 1 was “never” and 5 was “always”), how anxious, concerned, afraid and panicky they felt about their pregnancy. Maternal anxiety was assessed at 20 weeks of pregnancy using the State Anxiety Scale [21], a widely used maternal self-report measure. Postnatal maternal anxiety was assessed using the same measure at 9 weeks, 14 months, and 3.5 years to control for postnatal effects. The standardized mean across the three assessment points was used as the index of postnatal exposure to maternal anxiety. Maternal depression was assessed by self report using the Edinburgh Postnatal Depression Scale [22] at 3.5 years and was included in analyses to control for possible biasing effects on maternal reports of child behavioral and emotional symptoms.

### Assessment of maternal stroking

Maternal stroking was assessed by self report using the Parent–Infant Caregiving Touch Scale (PICTS) [23]. Four stroking items assessed how often (1 = never, 2 = rarely, 3 = sometimes, 4 = often, 5 = a lot) mothers currently stroked their baby’s face, back, tummy, arms, and legs. Reports made at 5 and 9 weeks correlated *r* = 0.58. As previously reported, there was no association between maternal stroking and maternal sensitivity assessed at 29 weeks, and the specificity of the effect of stroking was supported by the finding that breast feeding, which also entails skin to skin contact, did not predict the physiological and behavioral outcomes (14). We used the measure completed at 9 weeks of age because we had stroking data on 865 at that time point, in contrast to the 5 weeks measure that was available only from the intensive sample (*N* = 280).

### Child internalizing and externalizing symptoms

Maternal report of child symptoms was assessed at 3.5 years using the Preschool Child Behavior Checklist (CBCL) which has been extensively employed in studies of child and adolescent emotional and behavioral disorders [24]. It has 99 items each scored 0 (not true), 1 (somewhat or sometimes true), and 2 (very true or often true), which are summed to create seven syndrome scales, emotionally reactive, anxious/depressed, somatic complaints, withdrawn, sleep problems, attention problems, and aggressive behavior. An internalizing grouping total is generated by summing the emotionally reactive, anxious/depressed, somatic complaints and withdrawn scores, and an externalizing total by summing attention problems and aggressive behavior scores. To limit the number of analyses, and guided by previous animal and human evidence, initial analyses were conducted of anxious depressed, aggression and attentional syndrome scores, and total internalizing and externalizing scores. Raw scores were used throughout.

### Assessment of covariates

Demographic and biological risks known to be associated with prenatal stressors and child mental health disorders [25] were included as potential confounders. Variables generated at 20 weeks of pregnancy included mother’s age, her cohabiting/marital status, and whether or not she had stayed in education beyond 18 years. Socioeconomic status was determined using the revised English Index of Multiple Deprivation (IMD) [26] based on data collected from the UK Census in 2001. According to this system, postcode areas in England are ranked from most deprived (i.e. IMD of 1) to least deprived (i.e. IMD of 32,482) based on neighbourhood deprivation in seven domains: income, employment, health, education and training, barriers to housing and services, living environment, and crime. All mothers were given IMD ranks according to the postcode of the area where they lived and assigned to a quintile based on the UK distribution of deprivation. Variables for drinking alcohol and smoking in pregnancy were derived from information obtained at 20 weeks gestation. Birth records were used to determine sex of infant, birth weight by gestational age as a measure of fetal growth, and obstetric risk. Obstetric risk was rated using a weighted severity scale developed by a collaboration of American and Danish obstetricians and pediatric neurologists [27]. The scale has 32 items each of which has an assigned score in the range 1–5, and the highest rated item provides the value for analyses. It has been used widely in studies of perinatal complications and later development.

### Statistical analyses

To account for sample attrition from this general population sample up to the assessment at 3.5 years, we used inverse probability weights. Weights took account of identified factors associated with drop out; mothers’ age and years of education and maternal smoking. Variation in the weights associated with the covariates of each model was removed to improve efficiency.

All analyses were undertaken in Stata 12 [28]. Test statistics for weighted means and regression estimates were based on survey adjusted Wald tests (*t* tests if single degrees of freedom (*df*) or *F* tests if multiple *df*), using the robust ‘sandwich’ estimator of the parameter covariance matrix [29]. To account for the non-normal distribution of CBCL scores, only modest in the case of the internalizing and externalizing scores but more substantial for the anxiety-depression and hyperactivity-attention subscales, we used a model in which the expected residual variance was made a function of the mean. This was estimated using an iterative procedure in gllamm [30]; (http://www.gllamm.org) for estimating heteroscedastic linear regressions estimated by maximum likelihood in which the log-standard deviation of the Gaussian error was a linear function of the predicted mean, a generalization of the mean–variance equality assumed in Poisson regression. We began analyses with simple models that accounted for child age and gender as covariates and then proceeded to estimates from models that also covaried for the full set of confounders. We then explored the evidence for sex differences in effects. Following Little et al. [31], we centered and residualized the variables involved in the interactions, and their product that formed the interaction terms, to enable lower order interaction and main effects to remain interpretable as average effects. Interaction terms between statistical models were compared using postestimation Wald tests of equality.

## Results

### Characteristics of babies and mothers: preliminary analyses

Table 1 shows population estimates for the major variables in the analysis. The mean age of the mothers was 26.9 year (sd 5.9, range 18–51 years) and 75 % were either married or cohabiting. In this extensive sample 41.0 % were in the most deprived quintile of UK neighbourhoods [20], consistent with high levels of deprivation in some parts of the Wirral. A total of 48 women in the extensive sample (3.9 %) described themselves as other than White British.

**Table 1:** Summary of study variables *N* = 813

Assessment period	Measure	
20 weeks gestation	Maternal age, years [means(SD)]	26.87 (5.88)
	IMDQuintiles (%)	
	1 (most deprived)	333 (41.0)
	2–5	480 (59.0)
	Marital status (%)	
	Cohabiting	303 (37.3)
	Married	312 (38.4)
	Smoking status (%)	
	Pre pregnancy	158 (19.4)
	During pregnancy	159 (19.6)
	Higher education (%)^a^	471 (58.0)
	Alcohol during pregnancy (%)	195 (24.0)
	General State Anxiety [means(SD)]	31.61 (10.30)
	Pregnancy-specific Anxiety [means(SD)]	5.63 (3.26)
Birth	Obstetric Risk Weighting [means(SD)]	2.20 (1.17)
	Birth weight by gestational age, grams/week [means(SD)]	84.80 (11.88)
	Baby gender, males (%)	390 (48.0)
9 weeks postnatal	Mean stroking score [means(SD)]	3.89 (0.74)
3.5 years postnatal	CBCL internalizing, *R* score [means(SD)]	6.65 (5.58)
	CBCL anxious/depressed, *R* score [means(SD)]	1.64 (1.73)
	CBCL externalizing, *R* score [means(SD)]	9.77 (7.50)
	CBCL aggressive behaviors, *R* score [means(SD)]	7.70 (6.05)
	CBCL attention problems, *R* score [means(SD)]	2.06 (1.97)
	Maternal depression, EPDS [means(SD)]	5.21 (4.54)
	Age of child, months [means(SD)]	41.88 (2.36)
	Postnatal anxiety [means(SD)]^b^	0.045 (0.81)

Summaries are given as weighted averages based on the intensive allocation

*CBCL* Child Behavior Checklist

^a^Continuing education past 18 years of age

^b^Standardized mean variable (9 weeks to 3.5 years)

Online Data Supplement Table 1 shows associations between continuous variables and Online Data Supplement Table 2 those between categorical variables. Pregnancy-specific anxiety (PSA) and general state anxiety (GSA) were moderately correlated with each other, and with all of the CBCL outcomes. PSA and GSA were also associated with postnatal anxiety, and with social deprivation, single parent status, and smoking in pregnancy. In turn, many of the potential confounders were associated with each other and with the CBCL outcomes.

### Prenatal anxiety, maternal stroking, and CBCL internalizing symptoms

The main effects and interaction between maternal stroking and pregnancy-specific anxiety in the regression model predicting CBCL internalizing symptoms are shown in Table 2. The top rows show the results of analyses controlling for child sex and age, and lower rows show the effect of adjusting for the wider set of potential confounders. There was a highly significant main effect of PSA on child internalizing symptoms that was substantially reduced after adjusting for confounders. However, the interaction between PSA and maternal stroking, indicating moderation of PSA by maternal stroking, was significant prior to adjustment, and remained unchanged after addition of potential confounders. There was no indication of a sex difference in the interactions, and the test of the three-way interaction, PSA by stroking by sex of child was entirely non-significant. The effect of maternal stroking can be seen in Fig. 1 which contrasts children in low, medium, and high tertiles for maternal stroking. The figure shows locally weighted scatterplot smoothing (LOWESS) plots [32] fitted to the raw CBCL data, and the regression lines. It can be seen that the effect of increasing pregnancy-specific anxiety predicting increasing internalizing symptoms is greatest in the low stroking group, and becomes progressively weaker across the medium and high stroking groups. The regression models of CBCL anxious-depressed symptoms on PSA and maternal stroking are summarized in Online Supplementary Data Table 3. The main effect of PSA on anxious-depressed symptoms was similar to that for internalizing symptoms, being highly significant (*p* < 0.001) prior to, but non-significant (*p* = 0.215) following, adjustment for confounders. By contrast with internalizing symptoms, the PSA by maternal stroking interaction term was non-significant prior to (*p* = 0.311) and after (*p* = 0.282) controlling for confounders. The test of the three-way interaction, PSA by stroking by sex of child was entirely non-significant.

**Table 2:** Summary of regression analyses showing associations between 20 weeks prenatal pregnancy-specific anxiety, maternal stroking at 9 weeks, and internalizing CBCL scores at 3.5 years

3.5 years CBCL internalizing *R* score	Model 1 (pooled)		Model 2 (girls)		Model 3 (boys)	
	Coeff (SE)^a^	*p* value	Coeff (SE)^a^	*p* value	Coeff (SE)^a^	*p* value
Unadjusted						
PSA	1.077 (0.198)	0.000	1.018 (0.249)	0.000	1.124 (0.308)	0.000
Stroking	−0.064 (0.205)	0.754	−0.075 (0.234)	0.749	−0.026 (0.368)	0.944
PSA × stroking	−**0.450 (0.201)**	**0.026**	−**0.434 (0.219)**	**0.047**	−**0.528 (0.385)**	**0.170**
3.5 year child age	0.099 (0.095)	0.300	0.076 (0.096)	0.426	0.120 (0.172)	0.486
Gender	1.055 (0.389)	0.007				
Adjusted						
PSA	0.315 (0.167)	0.060	0.320 (0.219)	0.143	0.303 (0.245)	0.216
Stroking	−0.221 (0.175)	0.206	−0.239 (0.230)	0.298	−0.228 (0.255)	0.373
PSA × stroking	−**0.422 (0.164)**	**0.010**	−**0.371 (0.217)**	**0.087**	−**0.480 (0.265)**	**0.070**
3.5 year child age	0.029 (0.073)	0.692	0.035 (0.096)	0.711	−0.006 (0.111)	0.955
Gender	0.697 (0.324)	0.031				
Maternal age	−0.045 (0.032)	0.166	−0.041 (0.044)	0.347	−0.060 (0.045)	0.178
IMD quintiles	−0.063 (0.133)	0.633	0.021 (0.190)	0.911	−0.241 (0.177)	0.174
Marital status: cohabit	−1.750 (0.542)	0.001	−1.594 (0.614)	0.009	−1.958 (0.889)	0.028
Marital status: married	−0.799 (0.591)	0.176	−0.495 (0.665)	0.456	−1.043 (0.950)	0.272
Smoking status: previous	−0.012 (0.408)	0.976	−0.194 (0.533)	0.716	0.247 (0.607)	0.684
Smoking status: pregnancy	0.416 (0.522)	0.426	0.964 (0.676)	0.154	−0.562 (0.738)	0.446
Higher education	−0.890 (0.362)	0.014	−0.436 (0.456)	0.339	−1.610 (0.578)	0.005
Alcohol in pregnancy	−0.027 (0.379)	0.944	−0.540 (0.471)	0.252	0.268 (0.574)	0.641
Obstetric risk	−0.109 (0.142)	0.444	0.061 (0.181)	0.736	−0.328 (0.221)	0.138
BW/GA	−0.030 (0.014)	0.027	−0.034 (0.019)	0.077	−0.021 (0.021)	0.308
3.5 year depression	1.115 (0.224)	0.000	0.711 (0.285)	0.013	1.917 (0.350)	0.000
Postnatal anxiety	0.521 (0.289)	0.072	0.729 (0.375)	0.052	−0.058 (0.415)	0.889

The table shows coefficient (^a^robust standard errors) and significance for the effect of prenatal pregnancy-specific anxiety and the mean of 9 weeks maternal stroking, with an interaction (shown in bold) of main effects, accounting for conditional weighting in the models

*Models* model 1: main effects and interaction, model 2: main effects and interaction—girls, model 3: main effects and interaction—boys. Main effects made interpretable in presence of the interaction by means of orthogonalization [31]

*Variables* variables standardized: 20 weeks gestation anxiety, 9 weeks mean stroking, 3.5 years maternal depression, obstetric risk, mean maternal postnatal anxiety

Interactions: replaced by residuals from regression against other model covariates [31]

*PSA* pregnancy-specific anxiety, *BW/GA* birth weight by gestational age

As the PSA by stroking interaction predicted total internalizing, but not anxious-depressed symptoms, we conducted secondary analyses with the other three CBCL internalizing subscales, emotional reactivity, somatic symptoms, and social withdrawal, as dependent variables. Prior to inclusion of the full set of confounders, the interaction terms were (standardized coefficients (95 % confidence intervals): emotional reactivity −0.188 (−0.361, −0.015) *p* = 0.033, somatic −0.106 (−0.295, 0.084) *p* = 0.274, withdrawn − 0.111 (−0.225, 0.003) *p* = 0.056. Inclusion of confounders yielded similar results: emotional reactivity −0.142 (−0.293, 0.010) *p* = 0.067, somatic − 0.109 (−0.279, 0.060) *p* = 0.205, withdrawn − 0.106 (−0.207, −0.005) *p* = 0.040. Three-way interactions with sex of child were entirely non-significant for all three scales. There was a strong effect of generalized state anxiety (GSA) at 20 weeks on internalizing symptoms (*p* < 0.001) which was completely lost after accounting for confounders (*p* =  0.914) (Online Supplementary Data Table 4). The postnatal maternal anxiety and 3.5 year depression accounted for this difference. There was a similar, although smaller, contrast in the effect on anxious-depressed symptoms before and after adjustment for confounders. The GSA by stroking interactions was entirely non-significant. When compared using a test of postestimation coefficients, the sizes of the PSA × stroking, and GSA × stroking interactions predicting internalizing symptoms were significantly different (*p* = 0.0014).

### Prenatal anxiety, maternal stroking, and CBCL externalizing symptoms

The main effects and interaction between maternal stroking and PSA in the regression model predicting CBCL externalizing symptoms are shown in Table 3. The main effect of PSA remained after adjustment for confounders, and so did the PSA by stroking interaction. Figure 1 shows that pregnancy-specific anxiety was associated with externalizing symptoms only in the children of low stroking mothers, indicating moderation of PSA by maternal stroking. Although the interaction was somewhat stronger among girls than boys, the three way, sex of child by stroking by PSA interaction was not significant (*p* = 0.212). The summary of the regression models for CBCL aggression are shown in Online Supplementary Data Table 3. The main effect of PSA was highly significant prior to (*p* < 0.001) and following (*p* = 0.001) the inclusion of potential confounder variables. The PSA by maternal stroking interaction was significant in the unadjusted model (*p* = 0.007) and after adjusting for confounders (*p* = 0.002). The effect of the interaction on aggression was very similar to that shown in Fig. 1 for externalizing symptoms. There was some indication that the PSA by stroking interaction was stronger in girls than in boys but a test of the sex by PSA by stroking interaction was entirely non-significant. There were neither main nor interactive effects of PSA on attentional symptoms.

**Table 3:** Summary of regression analyses showing associations between 20 weeks prenatal pregnancy-specific anxiety, maternal stroking at 9 weeks, and externalizing CBCL scores at 3.5 years

3.5 years CBCL externalizing *R* score	Model 1 (pooled)		Model 2 (girls)		Model 3 (boys)	
	Coeff (SE)^a^	*p* value	Coeff (SE)^a^	*p* value	Coeff (SE)^a^	*p* value
Unadjusted						
PSA	1.849 (0.274)	0.000	1.636 (0.367)	0.000	2.233 (0.452)	0.000
Stroking	−0.196 (0.276)	0.478	−0.309 (0.338)	0.361	0.022 (0.460)	0.962
PSA × stroking	−**0.693 (0.282)**	**0.014**	−**0.907 (0.312)**	**0.004**	−**0.238 (0.526)**	**0.652**
3.5 year child age	0.038 (0.103)	0.709				
Gender	2.383 (0.523)	0.000				
Adjusted						
PSA	0.818 (0.245)	0.001	0.695 (0.292)	0.018	1.149 (0.405)	0.005
Stroking	−0.238 (0.238)	0.318	−0.435 (0.304)	0.152	−0.115 (0.373)	0.758
PSA × stroking	−**0.672 (0.236)**	**0.004**	−**0.791 (0.271)**	**0.004**	−**0.634 (0.427)**	**0.137**
3.5 year child age	−0.074 (0.091)	0.412	0.093 (0.121)	0.443	−0.253 (0.138)	0.067
Gender	1.868 (0.453)	0.000				
Maternal age	−0.075 (0.046)	0.104	−0.030 (0.060)	0.623	−0.111 (0.065)	0.089
IMD quintiles	−0.055 (0.173)	0.752	−0.134 (0.229)	0.558	−0.057 (0.254)	0.822
Marital status: cohabit	−1.519 (0.700)	0.030	−1.051 (0.862)	0.222	−2.095 (1.125)	0.063
Marital status: married	−0.625 (0.722)	0.386	−0.631 (0.899)	0.483	−0.874 (1.149	0.447
Smoking status: previous	1.193 (0.580)	0.040	0.999 (0.736)	0.174	1.568 (0.931)	0.092
Smoking status: pregnancy	2.156 (0.786)	0.006	1.947 (0.951)	0.041	2.023 (1.233)	0.101
Higher education	−0.865 (0.507)	0.088	0.221 (0.591)	0.709	−2.363 (0.848)	0.005
Alcohol in pregnancy	−0.429 (0.514)	0.403	−1.317 (0.609)	0.031	0.529 (0585)	0.537
Obstetric risk	−0.285 (0.197)	0.149	−0.326 (0.245)	0.183	−0.250 (0.306)	0.413
BW/GA	−0.023 (0.019)	0.227	−0.043 (0.023)	0.059	−0.001 (0.031)	0.990
3.5 year depression	1.063 (0.311)	0.001	0.921 (0.382)	0.016	1.239 (0.481)	0.010
Postnatal anxiety	0.634 (0.402)	0.115	0.568 (0.504)	0.259	0.576 (0.634)	0.364

The table shows coefficient (^a^robust standard errors) and significance for the effect of prenatal pregnancy-specific anxiety and the mean of 9 weeks maternal stroking, with an interaction (shown in bold) of main effects, accounting for conditional weighting in the models

*Models* model 1: main effects and interaction, model 2: main effects and interaction—girls, model 3: main effects and interaction—boys. Main effects made interpretable in presence of the interaction by means of orthogonalization [31]

*Variables* variables standardized: 20 weeks gestation anxiety, 9 weeks mean stroking, 3.5 years depression, obstetric risk, mean postnatal anxiety

Interactions: replaced by residuals from regression against other model covariates [31]

*PSA* pregnancy-specific anxiety, *BW/GA* birth weight by gestational age

Online Supplementary Data Table 4 shows the models predicting externalizing symptoms from GSA. There were strong main effects of GSA on CBCL externalizing symptoms (*p* < 0.001), aggression, (*p* < 0.001) and attentional symptoms (*p* < 0.001) prior to inclusion of confounders. After the addition of confounders the association with externalizing (*p* = 0.579), aggression (*p* = 0.543) and attentional (*p* = 0.918) symptoms became entirely nonsignificant. None of the GSA by stroking interactions was significant. There was no evidence for a difference of the anxiety measures, PSA, and GSA, in interaction with maternal stroking on the Externalizing symptoms (*p* = 0.188).

## Discussion

The analyses conducted in this paper were based on predictions from animal models which find that the fetal programing effects of prenatal stress on postnatal behaviors are mediated via decreased GR gene expression, and those, in the reverse direction, of postnatal licking and grooming and arched back nursing via increased GR gene expression. In humans, if tactile stimulation, in the form of maternal stroking soon after birth, has an effect similar to that of maternal behaviors on GR gene expression in rodents, it should substantially reduce the effect of an index of prenatal stress such as maternal anxiety. Having previously reported such an effect on outcomes at 29 weeks (*N* = 271) and 2.5 years (*N* = 243), we examined whether this persists to 3.5 years and whether it is seen in a much larger sample (*N* = 813). We found that frequency of infant stroking, assessed via maternal self report at when infants were on average 9-weeks-old, modified associations between pregnancy-specific anxiety at 20 weeks gestation and maternal ratings of internalizing, externalizing, and aggressive behaviors in children aged 3.5 years.

In this, the third report from the same study of the moderating effect of maternal stroking on infancy and early childhood outcomes, both remarkable similarities and important differences need to be brought out. There are three key similarities. First, the direction of effect of maternal stroking was the same on each occasion, such that an effect of the prenatal risk on child outcomes was markedly reduced for mothers who reported high levels of stroking. Second, the findings were in line with predictions based on the epigenetic effects of prenatal stress and tactile stimulation shown in animals. Third, the associations with prenatal risks were evident after controlling for postnatal exposures and a range of other possible confounders. Thus, we now have evidence from a large sample, of a specific, enduring effect of higher levels of maternal stroking to reduce the strength of association between a prenatal maternal risk and child emotional and behavioral symptoms.

Nevertheless, there were contrasts with the findings at 2.5 years, in that stroking modified the effect of pregnancy specific, but not general, anxiety, and there were no convincing sex differences. This is consistent with studies referred to earlier those likewise found predictions from pregnancy specific, but not general, anxiety. It is not clear, however, whether different psychological or biological processes are associated with pregnancy specific, contrasted with general, anxiety in pregnancy, or whether findings are better explained by measurement differences. Findings of effects of pregnancy-specific anxiety during the second, but not third trimester of pregnancy [7], and of general anxiety during the third, but not second trimester [5] might indicate different underlying processes. Equally, it could be that earlier in pregnancy measures of general anxiety reflect a mix of pre-pregnancy and pregnancy anxieties while pregnancy-specific anxiety measures, because of their focus, assess only recent or current anxieties. Later in pregnancy, by contrast, as the time since conception has increased, measures of general anxiety may be more likely to reflect symptoms that have been present only during pregnancy. We were unable to explore this further in this study, as we did not use a measure of pregnancy-specific anxiety at the third trimester 32 weeks assessment.

While none of the three-way interactions of sex of child by pregnancy-specific anxiety by maternal stroking was significant, there was modest power for these analyses, and it is not possible to conclude that they were absent. In particular, the two-way interactions for externalizing and aggression scores were significant only in girls. Nevertheless, the sex difference was much less evident than at 2.5 years. It could therefore be that the sex difference attenuates over time. Findings from this cohort with 29 weeks infancy outcomes do not, however, provide consistent evidence either way. We did not find a sex difference in the effect of maternal stroking on vagal withdrawal and temperament at this age [18], however, associations between prenatal anxiety and vagal withdrawal, and between low birth weight and vagal withdrawal were in opposite directions and the interaction terms were significant [33]. Furthermore, sex differences in associations between prenatal risks and later psychopathology over much longer periods of time have been reported [14–17]. A task remains to fit these fragments together into a single coherent description spanning the early development of boys and girls that would identify stable differences, and differential maturational leads and lags and patterns of expression.

### Strengths and limitations

The strengths of the study include the epidemiological design and the prospective measurement of anxiety during pregnancy, maternal stroking during early infancy, and behavioral outcomes at 3.5 years. Postnatal measurement of maternal anxiety on three occasions provided a robust test of the specificity of the prenatal effect. This was also examined in the presence of eight potential confounders, and current maternal depression to control for possible biasing effects of maternal mood.

We used maternal report of stroking as it draws on behavior that spans contexts in a way that experimental or naturalistic observation of a large community sample could not. We have previously reported support for its construct validity [19] and further support will require additional findings consistent with predictions based on the biology of early development. In the future, demonstrating agreement with observational measures will also be relevant to establishing validity, although such observational measures are generally limited in studies of human development by restricted coverage over place and time, and so cannot straightforwardly be considered as ‘gold standard’. As in the case of temperament research in infancy, in the absence of an agreed gold standard, self report and observational measures perform complementary functions [34]. A limitation of the study is that all of the measures were based on maternal report. Main effects may have arisen from shared reporting biases over the time points, although these could not explain systematic differences in associations linked to levels of stroking. Nevertheless, analyses based on information from other informants may not yield similar results.

### Implications

It remains to be seen whether the effects of maternal stroking in infancy described here, and in our previous reports, are mediated via GR gene methylation, or methylation of other key genes. The study of patterns of methylation in humans is at a very early stage with limitations arising not only from questions about the relevance of the epigenetics of peripheral cells to CNS gene expression, but also lack of clear evidence of the key CpG sites to be examined. However, findings from this [35] and other studies [36, 37] have replicated associations between prenatal depression or anxiety, and GR gene (*NR3C1*) 1-F promoter methylation at one particular CpG site, 36, which was also identified in a recent meta-analysis [38]. Furthermore, we have shown that maternal stroking reverses the effects of prenataland postnatal maternal depression on *NR3C1* methylation [35]. Equally, in animal models the expression of other receptors, such as oxytocin and vasopressin, is altered by maternal licking and grooming, and these may be equally relevant to the associations reported here [39].

Although the rationale for investigating maternal stroking in this study was the animal evidence for epigenetic effects of tactile stimulation on HPA axis regulation, in humans tactile stimulation has many other effects. Characteristic patterns of prefrontal cortex and limbic activations have been shown in response to stroking with a pleasant stimulus, such as velvet, contrasted with a neutral or unpleasant stimulus, such as sandpaper [40]. Connectivity between these regions is central to effective emotional and behavioral regulation, and impaired functioning of each region and failures of connectivity have been hypothesized to underpin conditions such as depression and borderline personality disorder [41]. It is possible that repeated stroking early in development, leading to activations in these regions, may enhance their functions and their connectivity.

Irrespective of the mechanism, we now have increasing evidence from this study that maternal stroking modifies the effects of indices of prenatal stress, such as maternal depression and anxiety, and over substantial periods of time. It remains to be seen whether these effects continue through childhood and beyond, whether they are modified by later experiences, and whether sex differences are evident at later follow-up. If replicated in other samples, there will be major implications for our understanding of the interplay between prenatal and postnatal influences, and ultimately for the development of treatments and services during pregnancy and early infancy.

## Electronic supplementary material

Below is the link to the electronic supplementary material.

Supplementary material 1 (DOC 211 kb)
