# Supplementary material for: Prenatal anxiety, maternal stroking in infancy, and symptoms of emotional and behavioral disorders at 3.5 years
Source: Eur Child Adolesc Psychiatry. 2016 Jul 27;26(3):325–34. doi: 10.1007/s00787-016-0886-6 (PMC5323471; doi:10.1007/s00787-016-0886-6)
Supplement: Supplementary file 1 — Supplementary material 1 (DOC 211 kb) [file 787_2016_886_MOESM1_ESM.doc]

**Online Data Supplement Table 1.** Correlation matrix for pre and post natal continuous variables

|  | Maternal | 20 weeks | 20 weeks | Obstetric | Birthweight | 9 weeks | 3.5 years | 3.5 years | 3.5 years | 3.5 years | 3.5 | 3.5 years | 3.5 years |
| --- | --- | --- | --- | --- | --- | --- | --- | --- | --- | --- | --- | --- | --- |
|  | Age | Gestation | Gestation | risk | by | Mean | Internalizing | Anxious/ | Externalising | Aggressive | Attentional | Maternal | Child |
|  |  | GSA | PSA |  | Gestational | Stroking |  | Depressed |  | Behaviours | Problems | depression | age |
|  |  |  |  |  | age |  |  |  |  |  |  |  |  |
| Maternal Age |  |  |  |  |  |  |  |  |  |  |  |  |  |
| 20 weeks gestation GSA | **-0.1301** | 1.0000 |  |  |  |  |  |  |  |  |  |  |  |
| 20 week gestation  PSA | **-0.0882** | **0.4657** | 1.0000 |  |  |  |  |  |  |  |  |  |  |
| Obstetric risk | **0.1248** | 0.0422 | 0.0678 | 1.0000 |  |  |  |  |  |  |  |  |  |
| Birthweight by gestational age | 0.0228 | -0.0682 | -0.0354 | 0.0038 | 1.0000 |  |  |  |  |  |  |  |  |
| 9 weeks  Mean Stroking | -0.0370 | -0.0342 | 0.0417 | 0.0384 | 0.0181 | 1.0000 |  |  |  |  |  |  |  |
| 3.5 years  Internalizing | **-0.1621** | **0.1413** | **0.1853** | 0.0133 | **-0.1187** | -0.0109 | 1.0000 |  |  |  |  |  |  |
| 3.5 years  Anxious / Depressed | **-0.0922** | **0.0845** | **0.1384** | 0.0446 | **-0.1301** | 0.0462 | **0.8019** | 1.0000 |  |  |  |  |  |
| 3.5 years  Externalizing | **-0.2088** | **0.1422** | **0.2294** | -0.0322 | **-0.0716** | -0.0113 | **0.6683** | **0.4218** | 1.0000 |  |  |  |  |
| 3.5 years  Aggressive Behaviours | **-0.1937** | **0.1349** | **0.2248** | -0.0488 | **-0.0788** | -0.0127 | **0.6670** | **0.4313** | **0.9807** | 1.0000 |  |  |  |
| 3.5 years  Attention Problems | **-0.2009** | **0.1276** | **0.1835** | 0.0271 | -0.0307 | -0.0040 | **0.4978** | **0.2827** | **0.7986** | **0.6653** | 1.0000 |  |  |
| 3.5 years  Maternal depression | **-0.1333** | **0.3499** | **0.2959** | 0.0249 | **-0.0720** | 0.0683 | **0.2894** | **0.2170** | **0.2884** | **0.2768** | **0.2488** | 1.0000 |  |
| 3.5 years  Child age | **-0.1192** | **0.0719** | 0.0601 | 0.0472 | -0.0495 | -0.0133 | 0.0651 | 0.0648 | 0.0103 | 0.0055 | 0.0225 | **0.0768** | 1.0000 |
| Postnatal maternal anxiety | **-0.1328** | **0.4684** | **0.3466** | 0.0580 | -0.0648 | -0.0085 | **0.2323** | **0.1652** | **0.2468** | **0.2397** | **0.2041** | **0.6277** | 0.0278 |

Correlations are given as weighted averages, point estimates from aweight. Significant correlations are shown in **bold,** estimates from pweight.

PSA pregnancy specific anxiety. GSA general state anxiety

**Online Data Supplement Table 2. Correlation Matrix ordered categorical and binary variables.**

|  | **Higher** | **Alcohol** | **Child** | **IMD** | **Marital** | **Smoking** |
| --- | --- | --- | --- | --- | --- | --- |
|  | **Education** | **Status** | **Gender** | **Quintiles** | **Status** | **Status** |
|  |  |  |  |  |  |  |
| Higher Education | 1.0000 |  |  |  |  |  |
| Alcohol Status | -0.0718 | 1.0000 |  |  |  |  |
| Child Gender | 0.0522 | -0.0127 | 1.0000 |  |  |  |
| IMD Quintiles | **0.2861** | -0.0324 | 0.0430 | 1.0000 |  |  |
| Marital Status | **0.4706** | 0.0058 | -0.0041 | **0.3707** | 1.0000 |  |
| Smoking Status | **-0.3739** | **0.2313** | -0.0018 | **-0.2886** | **-0.4515** | 1.0000 |
| Maternal Age | **0.3266** | 0.0385 | 0.0091 | **0.3337** | **0.5764** | **-0.3650** |
| 20 weeks gestation GSA | -0.0846 | 0.0534 | -0.0930 | **-0.0972** | **-0.2688** | **0.1874** |
| 20 weeks gestation PSA | -0.0817 | 0.0515 | -0.0353 | **-0.1019** | **-0.1477** | **0.2175** |
| Obstetric Risk | 0.0184 | 0.0317 | 0.0062 | **-0.1059** | 0.0778 | 0.0069 |
| Birth Weight by Gestational Age | 0.0758 | -0.0332 | **0.1555** | 0.0651 | **0.1355** | **-0.1587** |
| 9 weeks Mean Stroking | **-0.1258** | **-0.1874** | 0.0229 | -0.0047 | -0.0414 | 0.0293 |
| 3.5 years Internalizing | **-0.1775** | <0.0013 | **0.1259** | **-0.1230** | **-0.2062** | **0.1831** |
| 3.5 years Anxious/ Depressed | **-0.1991** | -0.0092 | 0.0389 | -0.0671 | **-0.1522** | **0.0921** |
| 3.5 years Externalizing | **-0.1861** | 0.0096 | **0.2011** | **-0.1260** | **-0.2250** | **0.2830** |
| 3.5 years Aggressive Behaviours | **-0.1634** | 0.0067 | **0.1776** | **-0.1051** | **-0.2033** | **0.2737** |
| 3.5 years Attention Problems | **-0.2062** | 0.0157 | **0.2191** | **-0.1561** | **-0.2329** | **0.2386** |
| 3.5 years Maternal Depression | -0.0721 | **0.1080** | 0.0574 | **-0.0807** | **-0.1621** | **0.1550** |
| 3.5 years Child’s Age | **-0.1322** | 0.0407 | 0.0319 | -0.0628 | **-0.0997** | 0.0628 |
| Postnatal Maternal Anxiety | **-0.0971** | **0.1063** | 0.0633 | -0.0646 | **-0.1982** | **0.1817** |

Tetrachoric , tetraserial, polychoric and polyserial correlations shown for; dichotomous; dichotomous and continuous; ordinal; ordinal and continuous variables respectively. Polychoric command in STATA allows all correlation types, aweighting applied. Significant correlations shown in **bold** and derived from individual regressions allowing for pweighting. Ordinal logistic and logistic regression utilised for ordinal and binary dependent variables.

PSA: pregnancy specific anxiety

GSA: general state anxiety

Smoking status: ordered categories, never smoked, smoked before pregnancy, smoked before and during pregnancy

Child gender: 0 = female, 1 = male

**Online Data Supplement Table 3. Summary of multivariate regression analyses showing associations between 20 weeks prenatal pregnancy-specific anxiety, maternal stroking at 9 weeks, and CBCL subscales, anxious depressed, attention problems and aggression scores, at 3.5 years**

|  | **Model 1 (pooled)** | | **Model 2 (girls)** | | **Model 3 (boys)** | | **Model 1 (pooled)** | | **Model 2 (girls)** | | **Model 3 (boys)** | | | | |
| --- | --- | --- | --- | --- | --- | --- | --- | --- | --- | --- | --- | --- | --- | --- | --- |
|  | **Coeff(SE^a^)** | **p-value** | **Coeff(SE)** | **p-value** | **Coeff(SE)** | **p-value** | **Coeff(SE)** | **p-value** | **Coeff(SE)** | **p-value** | **Coeff(SE)** | | | **p-value** | |
| **Unadjusted** | | | | | | | **Adjusted^b^** | | | | | | | | |
| **3.5 years CBCL Anxious / Depressed R-Score** | | | | | | | | | | | | | | | |
| **PSA** | 0.226 (0.060) | <0.001 | 0.251 (0.085) | 0.003 | 0.211 (0.088) | 0.016 | 0.077 (0.062) | 0.215 | 0.119 (0.085) | 0.159 | | 0.044 (0.089) | | 0.617 | |
| **Stroking** | 0.070 (0.065) | 0.285 | 0.048 (0.082) | 0.559 | 0.108 (0.106) | 0.308 | -0.014 (0.058) | 0.802 | -0.013 (0.083) | 0.873 | | -0.018 (0.080) | | 0.825 | |
| **PSA *Stroking** | -0.064 (0.063) | 0.311 | -0.027 (0.074) | 0.715 | -0.106 (0.105) | 0.311 | -0.061 (0.057) | 0.282 | -0.026 (0.076) | 0.729 | | -0.085 (0.083) | | 0.304 | |
| **3.5 year Child Age** | 0.033 (0.031) | 0.281 | 0.012 (0.035) | 0.735 | 0.056 (0.048) | 0.245 | 0.018 (0.024) | 0.441 | 0.011 (0.034) | 0.755 | | 0.020 (0.033) | | 0.544 | |
| **Gender** | 0.097 (0.123) | 0.432 |  |  |  |  | 0.060 (0.110) | 0.590 |  |  | |  | |  | |
| **3.5 years CBCL Attention Problems R-Score** | | | | | | | | | | | | | | | |
| **PSA** | 0.370 (0.069) | <0.001 | 0.291 (0.099) | 0.003 | 0.504 (0.107) | <0.001 | 0.138 (0.065) | 0.034 | 0.115 (0.085) | 0.176 | | | 0.241 (0.090) | 0.007 | |
| **Stroking** | -0.024 (0.073) | 0.748 | -0.004 (0.094) | 0.969 | -0.025 (0.118) | 0.831 | -0.070 (0.062) | 0.262 | -0.067 (0.074) | 0.363 | | | -0.112 (0.101) | 0.265 | |
| **PSA *Stroking** | -0.076 (0.075) | 0.314 | -0.142 (0.094) | 0.131 | 0.069 (0.119) | 0.560 | -0.076 (0.068) | 0.267 | -0.138 (0.081) | 0.087 | | | -0.073 (0.104) | 0.484 | |
| **3.5 year Child Age** | 0.021 (0.028) | 0.454 | 0.068 (0.035) | 0.050 | -0.032 (0.048) | 0.502 | -0.006 (0.024) | 0.789 | 0.052 (0.030) | 0.085 | | | -0.057 (0.034) | 0.095 | |
| **Gender** | 0.683 (0.136) | <0.001 |  |  |  |  | 0.584 (0.119) | <0.001 |  |  | | |  |  | |
| **3.5 years CBCL Aggressive Behaviours R-Score** | | | | | | | | | | | | | | | |
| **PSA** | 1.468 (0.223) | <0.001 | 1.320 (0.293) | <0.001 | 1.723 (0.365) | <0.001 | 0.675 (0.205) | 0.001 | 0.590 (0.243) | 0.015 | | 0.905 (0.344) | | | 0.008 |
| **Stroking** | -0.170 (0.223) | 0.446 | -0.306 (0.273) | 0.262 | 0.048 (0.371) | 0.897 | -0.175 (0.198) | 0.376 | -0.378 (0.260) | 0.146 | | -0.004 (0.301) | | | 0.990 |
| **PSA *Stroking** | -0.609 (0.227) | 0.007 | -0.759 (0.250) | 0.002 | -0.314 (0.431) | 0.466 | -0.592 (0.195) | 0.002 | -0.686 (0.255) | 0.002 | | -0.552 (0.354) | | | 0.119 |
| **3.5 year Child Age** | 0.013 (0.085) | 0.876 | 0.084 (0.095) | 0.375 | -0.086 (0.151) | 0.568 | -0.073 (0.076) | 0.336 | 0.033 (0.101) | 0.744 | | -0.199 (0.117) | | | 0.088 |
| **Gender** | 1.713 (0.422) | <0.001 |  |  |  |  | 1.312 (0.373) | <0.001 |  |  | |  | | |  |

The table shows coefficient (^a^robust standard errors) and significance for the effect of prenatal pregnancy-specific anxiety and the mean of 9 weeks maternal stroking, with an interaction of main effects, accounting for conditional weighting in the models. ^b^Adjusted models covary for confounding variables.

**Models**

Model 1: Main effects and interaction, Model 2: Main effects and interaction –girls , Model 3: Main effects and interaction – boys. Main effects made interpretable in presence of the interaction by means of orthogonalization (31).

**Variables**

Variables standardised: 20 weeks gestation anxiety, 9 weeks mean stroking , 3.5 years depression, obstetric risk, mean post natal anxiety

Interactions: Replaced by residuals from regression against other model covariates [31]

**Confounders**

Maternal: Age, marital status (cohabiting / married / single), higher education, IMD Quintiles, smoking status (before pregnancy, during pregnancy, never), alcohol in pregnancy, obstetric risk, 3.5 years depression, mean post natal anxiety

Paediatric: Birth weight by gestation age, exact age at 3.5 years data collection, gender.

PSA Pregnancy-Specific Anxiety

**Online Data Supplement Table 4. Summary of multivariate regression analyses showing associations between 20 weeks general state anxiety, maternal stroking at 9 weeks, and CBCL subscales at 3.5 years**

|  | **Model 1 (pooled)** | | **Model 2 (girls)** | | **Model 3 (boys)** | | **Model 1 (pooled)** | | | **Model 2 (girls)** | | **Model 3 (boys)** | | | | |
| --- | --- | --- | --- | --- | --- | --- | --- | --- | --- | --- | --- | --- | --- | --- | --- | --- |
|  | **Coeff(SE^a^)** | **p-value** | **Coeff(SE)** | **p-value** | **Coeff(SE)** | **p-value** | **Coeff(SE)** | | **p-value** | **Coeff(SE)** | **p-value** | **Coeff(SE)** | | | **p-value** | |
| **Unadjusted** | | | | | | | **Adjusted^b^** | | | | | | | | | |
| **3.5 years CBCL Internalizing R-Score** | | | | | | | | | | | | | | | | |
| **GSA** | 0.927 (0.227) | <0.001 | 0.586 (0.251) | 0.020 | 1.301 (0.434) | 0.003 | -0.021 (0.195) | | 0.914 | -0.294 (0.242) | 0.224 | | | 0.448 (0.317) | 0.158 | |
| **Stroking** | -0.001 (0.210) | 0.995 | -0.057 (0.245) | 0.817 | 0.131 (0.358) | 0.713 | -0.147 (0.178) | | 0.410 | -0.213 (0.233) | 0.361 | | | 0.003 (0.267) | 0.991 | |
| **GSA *Stroking** | 0.237 (0.216) | 0.274 | -0.011 (0.231) | 0.961 | 0.613 (0.454) | 0.177 | 0.083 (0.182) | | 0.648 | -0.016 (0.221) | 0.944 | | | 0.447 (0.309) | 0.148 | |
| **3.5 year Child Age** | 0.108 (0.084) | 0.196 | 0.112 (0.100) | 0.266 | 0.115 (0.141) | 0.416 | 0.045 (0.072) | | 0.528 | 0.059 (0.095) | 0.534 | | | 0.001 (0.109) | 0.996 | |
| **Gender** | 1.044 (0.392) | 0.008 |  |  |  |  | 0.679 (0.331) | | 0.040 |  |  | | |  |  | |
| **3.5 years CBCL Anxious / Depressed R-Score** | | | | | | | | | | | | | | | | |
| **GSA** | 0.178 (0.073) | 0.015 | 0.052 (0.087) | 0.547 | 0.329 (0.115) | 0.004 | -0.037 (0.068) | | 0.588 | -0.175 (0.085) | 0.040 | | 0.118 (0.105) | | 0.261 | |
| **Stroking** | 0.087 (0.067) | 0.191 | 0.055 (0.082) | 0.502 | 0.151 (0.105) | 0.152 | -0.002 (0.058) | | 0.971 | -0.008 (0.082) | 0.919 | | 0.030 (0.083) | | 0.718 | |
| **GSA *Stroking** | 0.100 (0.071) | 0.162 | 0.053 (0.082) | 0.521 | 0.196 (0.128) | 0.125 | 0.038 (0.059) | | 0.521 | 0.022 (0.073) | 0.759 | | 0.106 (0.091) | | 0.247 | |
| **3.5 year Child Age** | 0.038 (0.028) | 0.167 | 0.035 (0.036) | 0.336 | 0.055 (0.042) | 0.193 | 0.023 (0.024) | | 0.327 | 0.020 (0.034) | 0.185 | | 0.020 (0.032) | | 0.528 | |
| **Gender** | 0.063 (0.122) | 0.609 |  |  |  |  | 0.049 (0.113) | | 0.661 |  |  | |  | |  | |
| **3.5 years CBCL Externalizing R-Score** | | | | | | | | | | | | | | | | |
| **GSA** | 1.188 (0.269) | <0.001 | 0.931 (0.329) | 0.005 | 1.586 (0.484) | 0.001 | -0.137 (0.247) | 0.579 | | -0.134 (0.325) | 0.681 | | 0.124 (0.402) | | | 0.758 |
| **Stroking** | -0.067 (0.273) | 0.807 | -0.219 (0.344) | 0.525 | 0.208 (0.455) | 0.648 | -0.171 (0.241) | 0.479 | | -0.361 (0.303) | 0.234 | | -0.019 (0.384) | | | 0.961 |
| **GSA *Stroking** | -0.343 (0.263) | 0.192 | -0.432 (0.300) | 0.150 | -0.120 (0.499) | 0.810 | -0.345 (0.222) | 0.119 | | -0.429 (0.281) | 0.127 | | -0.267 (0.445) | | | 0.548 |
| **3.5 year Child Age** | 0.024 (0.106) | 0.820 | 0.183 (0.135) | 0.175 | -0.179 (0.171) | 0.294 | -0.063 (0.092) | 0.494 | | 0.129 (0.122) | 0.292 | | -0.253 (0.140) | | | 0.071 |
| **Gender** | 2.500 (0.537) | <0.001 |  |  |  |  | 1.811 (0.462) | <0.001 | |  |  | |  | | |  |
| **3.5 years CBCL Aggressive Behaviours R-Score** | | | | | | | | | | | | | | | | |
| **GSA** | 0.904 (0.215) | <0.001 | 0.688 (0.260) | 0.008 | 1.252 (0.390) | 0.001 | -0.122 (0.201) | | 0.543 | -0.132 (0.268) | 0.622 | | 0.083 (0.328) | | | 0.801 |
| **Stroking** | -0.065 (0.222) | 0.768 | -0.237 (0.280) | 0.398 | 0.203 (0.365) | 0.578 | -0.125 (0.200) | | 0.532 | -0.328 (0.258) | 0.204 | | 0.072 (0.308) | | | 0.816 |
| **GSA *Stroking** | -0.348 (0.210) | 0.097 | -0.436 (0.237) | 0.066 | -0.126 (0.399) | 0.752 | -0.340 (0.181) | | 0.061 | -0.392 (0.235) | 0.095 | | -0.258 (0.352) | | | 0.464 |
| **3.5 year Child Age** | 0.001 (0.086) | 0.989 | 0.115 (0.109) | 0.288 | -0.142 (0.140) | 0.309 | -0.063 (0.076) | | 0.410 | 0.066 (0.102) | 0.052 | | -0.201 (0.117) | | | 0.087 |
| **Gender** | 1.783 (0.434) | <0.001 |  |  |  |  | 1.252 (0.380) | | 0.001 |  |  | |  | | |  |
| **3.5 years CBCL Attenional Behaviours R-Score** | | | | | | | | | | | | | | | | |
| **GSA** | 0.283 (0.072) | <0.001 | 0.242 (0.092) | 0.008 | 0.320 (0.125) | 0.010 | -0.008 (0.075) | | 0.918 | -0.007 (0.101) | 0.942 | | | 0.045 (0.101) | 0.656 | |
| **Stroking** | -0.001 (0.072) | 0.989 | 0.005 (0.095) | 0.958 | 0.010 (0.118) | 0.934 | -0.056 (0.063) | | 0.376 | -0.044 (0.076) | 0.567 | | | -0.095 (0.102) | 0.351 | |
| **GSA *Stroking** | -0.005 (0.076) | 0.952 | -0.004 (0.092) | 0.966 | 0.005 (0.134) | 0.970 | -0.001 (0.069) | | 0.988 | -0.036 (0.080) | 0.655 | | | -0.019 (0.121) | 0.874 | |
| **3.5 year Child Age** | 0.019 (0.028) | 0.492 | 0.064 (0.036) | 0.077 | -0.038 (0.046) | 0.408 | -0.005 (0.024) | | 0.845 | 0.052 (0.031) | 0.088 | | | -0.055 (0.035) | 0.114 | |
| **Gender** | 0.731 (0.140) | <0.001 |  |  |  |  | 0.578 (0.121) | | <0.001 |  |  | | |  |  | |

The table shows coefficient (^a^robust standard errors) and significance for the effect of prenatal pregnancy-specific anxiety and the mean of 9 weeks maternal stroking, with an interaction of main effects, accounting for conditional weighting in the models. ^b^Adjusted models covary for confounding variables.

**Models**

Model 1: Main effects and interaction, Model 2: Main effects and interaction – girls , Model 3: Main effects and interaction – boys. Main effects made interpretable in presence of the interaction by means of orthogonalization (31).

**Variables**

Variables standardised: 20 weeks GSA, 9 weeks mean stroking, 3.5 years depression, obstetric risk, mean post natal anxiety

Interactions: Replaced by residuals from regression against other model covariates [31]

**Confounders**

Maternal: Age, marital status (cohabiting / married / single), higher education, IMD Quintiles, smoking status (before pregnancy, during pregnancy, never), alcohol in pregnancy, obstetric risk, 3.5 years depression, mean post natal anxiety

Paediatric: Birth weight by gestation age, exact age at 3.5 years data collection, gender.

GSA General State Anxiety

**Online Data Supplement Figure 1.** Flow diagram of recruitment from 20 weeks gestation to 3.5 years follow up

Postnatal maternal state anxiety is the mean of the state anxiety scores obtained at the 9 weeks, 14 months and 3.5 years assessments.
